# A potential therapeutic approach for gastric cancer: inhibition of LACTB transcript 1

**DOI:** 10.18632/aging.205345

**Published:** 2023-12-26

**Authors:** Wei Nie, Lihua Hu, Zhiqiang Yan, Yang Wang, Qianyun Shi, Shui He, Qian Wang, Fang Yang

**Affiliations:** 1 Center of Clinical Laboratories, The Affiliated Hospital of Guizhou Medical University, Guiyang, China; 2 Department of Basic Clinical Laboratory and Hematology, School of Clinical Laboratory Science, Guizhou Medical University, Guiyang, China; 3 Department of Gastrointestinal Surgery, Affiliated Hospital of Guizhou Medical University, Guiyang, China

**Keywords:** LACTB transcript 1, gastric cancer, adaptive immune resistance, stemness, precision medicine

## Abstract

Background: This study sought to investigate the role of LACTB transcript 1 in regulating adaptive immune resistance and stemness in gastric cancer and its potential as a therapeutic target for precision medicine.

Methods: Bioinformatics analysis and RT-qPCR were used to analyze the expression level of LACTB and its transcripts in gastric cancer cells. The effects of LACTB transcript 1 on adaptive immune resistance and stemness were evaluated using *in vitro* cell experiments and western blotting experiments.

Results: Our study findings revealed that LACTB transcript 1 modulated adaptive immune resistance and inhibited the stemness of gastric cancer cells. Knocking down the expression level of LACTB transcript 1 activated autophagy and inhibited EMT. As expected, overexpression of LACTB transcript 1 yielded the opposite findings. The expression level of LACTB transcript 1 in the peripheral blood of gastric cancer patients was consistent with the bioinformatics analysis, suggesting its potential as a biomarker of gastric cancer.

Conclusions: LACTB transcript 1 is a promising therapeutic target for precision medicine in gastric cancer by modulating immune evasion mechanisms and stemness. These findings provide insights into leveraging long non-coding RNAs (lncRNAs) in immunotherapy, radiotherapy, and chemotherapy to enhance cancer therapy efficacy, particularly in the context of targeting tumor heterogeneity and stemness.

## INTRODUCTION

Gastric cancer represents a major public health concern worldwide, with nearly 1.09 million new cases reported in 2020, ranking fifth in terms of incidence and fourth in terms of mortality globally [1]. The development of gastric cancer is closely associated with several factors, including family history, diet, alcohol consumption, smoking, as well as infections by Helicobacter pylori and Epstein-Barr virus [2]. Currently, the standard of care treatment for gastric cancer involves surgery, which is often supplemented by neoadjuvant chemotherapy. Although this treatment approach has demonstrated significant clinical efficacy [3], the survival rate for patients with advanced gastric cancer has not shown a significant improvement. Treatment failure is largely attributed to cancer cell metastasis, and it has been established that the migration and invasion of gastric cancer are closely associated with genetic factors. Accordingly, it is essential to identify novel gene markers and comprehensively comprehend their effects and mechanisms concerning the invasion and metastasis of gastric cancer cells. This will contribute to the development of effective diagnosis and treatment strategies for gastric cancer.

LACTB is a newly discovered tumor-related gene that has emerged in recent years, distributed across various human tissues [4], and is localized in the mitochondrial intermembrane space. It has been shown to exert an impact on the flux of metabolites at specific sites within the metabolic network [5]. Nina Peitsaro and her team of researchers have highlighted the significant role played by LACTB in the initiation and progression of cancer. LACTB can regulate mitochondrial lipid metabolism through mitochondrial phosphatidylserine decarboxylase, thus modulating the proliferation and differentiation of cancer cells [6]. An increasing body of evidence suggests that LACTB’s upstream regulation can be modulated by microRNAs, while its downstream activities can impact various functional factors, such as MCP-1, P53, PCNA, MMP9, MMP2, and VEGF [7–10]. Previous investigations have demonstrated that LACTB is notably downregulated in several types of cancer, including hepatocellular carcinoma [11], breast cancer [8], colorectal cancer [9, 12, 13], glioma [10], and melanoma [14]. Upregulating the expression of LACTB in the aforementioned tumors has been observed to mitigate the invasion and migration potential of tumor cells, exhibiting a tumor suppressor effect. However, recent research has revealed that LACTB expression levels are upregulated in nasopharyngeal carcinoma [15] and pancreatic cancer [16], and it can potentially enhance the invasive, migratory, and proliferative abilities of cancer cells. The above studies overlap in their assertion that the expression and mechanism of LACTB varies in different types of tumors.

In a previous study, gene enrichment analysis LACTBindicated LACTB was significantly associated with the lysosome function, which serves as the central hub for autophagy [17]. Autophagy is a highly coordinated process that facilitates the degradation of damaged or aging organelles, misfolded proteins, and pathogens via lysosomal degradation pathways [18]. Moreover, autophagy is believed to modulate nuclear events that safeguard genome stability, thereby potentially contributing to the prevention of tumorigenesis [19]. In the tumor microenvironment, LACTB can potentially suppress pro-tumor inflammatory signals and enhance the anti-cancer immunity of myeloid cells, thereby exerting an anti-tumor effect [20]. However, under metabolic stress, autophagy can promote tumorigenesis by providing nutrients to malignant tumor cells, thereby facilitating tumor cell survival [20–23]. As a result, autophagy can not only reduce the proliferation, migration, and invasion of gastric cancer cells [24] but also trigger autophagic cell death to suppress tumor progression [25]. Additionally, several studies have revealed a potential crosstalk between autophagy and epithelial-mesenchymal transition (EMT) in tumor cells, which may have dual effects on EMT [26]. On one hand, autophagy inhibitors have been observed to mitigate EMT in renal cancer cells [27]. On the other hand, elevating the expression of autophagy levels may reduce the expression of the EMT phenotype in glioblastoma cells [28]. Therefore, alterations in autophagy levels may be associated with the progression of EMT in cancer cells. EMT is recognized to play a critical role in the migration and invasion of tumor cells [29, 30] and is typically viewed as an initial key event that triggers tumor invasion and metastasis [29–31]. During EMT in cancer cells, the expression of cell adhesion molecules, such as E-cadherin, is downregulated, while stromal markers, including Vimentin and N-cadherin, are upregulated. This molecular event triggers cell migration and invasion [32], leading to local invasion, intravasation, and extravasation of tumor cells into the systemic circulation. Ultimately, this process facilitates the formation of early metastatic colonies at distant sites [33]. Over the years, numerous experts have confirmed that EMT is significantly associated with the metastasis of gastric cancer and can potentially enhance the migratory capability of gastric cancer cells by inducing the upregulation of NFE2L3 [34] or PADI4 [35].

In summary, LACTB could potentially impact the EMT of tumor cells by modulating autophagy, subsequently affecting the migratory and invasive potential of gastric cancer cells. Consequently, the present study sought to investigate the impact of LACTB on the expression levels of autophagy and EMT key proteins in gastric cancer cells by generating a gastric cancer cell model with LACTB overexpression or knockdown. Furthermore, our study sought to explore the mechanism underlying LACTB-mediated autophagy involved in the EMT of gastric cancer cells.

## RESULTS

### Bioinformatics analysis of LACTB

**Figure 1:**
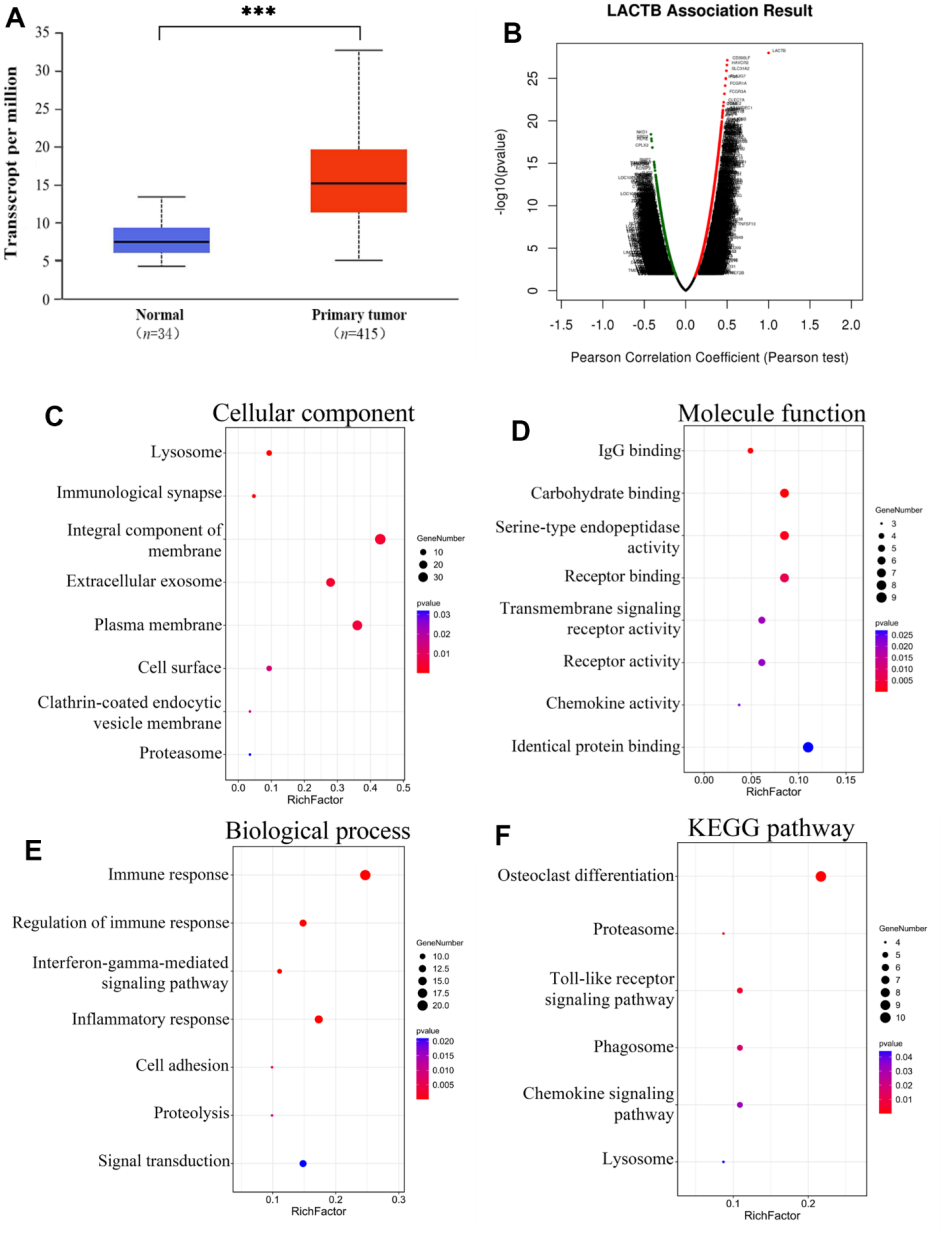
**Enrichment analysis of the genes altered in the LACTB neighborhood in gastric cancer.** (**A**) Expression of *LACTB* in gastric cancer tissue. (**B**) Pearson test was used to analyze correlations between LACTB and genes differentially expressed in gastric cancer. (**C**) Cellular components. (**D**) Molecular functions. (**E**) Biological processes. (**F**) KEGG pathway analysis. The rich factors are represented by the *p*-value and number of genes in the x-axis. ^***^P<0.001 vs. Normal.

Data from 449 specimens sourced from TCGA were obtained via the UALCAN bioinformatics analysis website, encompassing 34 normal controls and 415 individuals with gastric cancer. An examination of the data unveiled a notably elevated expression level of LACTB mRNA in gastric cancer tissues in comparison to normal controls (P<0.001) (Figure 1A). Functional modules of LinkedOmics were utilized to analyze the co-expressed genes of LACTB in gastric cancer (Supplementary Table 2), and the results are visualized in a volcano plot (Figure 1B). Significant genes with differential expression were selected for enrichment analysis. GO analysis revealed that LACTB-related genes were primarily localized in lysosomes, immune synapses, cell membranes, and other cellular structures (Figure 1C). It can participate in the formation of proteasome complexes and perform molecular functions via its receptor activity, chemokine activity, and binding properties to proteins and carbohydrates (Figure 1D). Furthermore, LACTB is involved in various biological processes, including immune response, inflammatory response, cell adhesion, signal transduction, and proteolysis (Figure 1E). KEGG pathway analysis demonstrated that LACTB-related genes are primarily involved in lysosome, proteasome, phagosome, and some cytokine signaling pathways (Figure 1F).

### Expression and diagnostic value of different transcripts of LACTB in peripheral blood of gastric cancer patients

**Figure 2:**
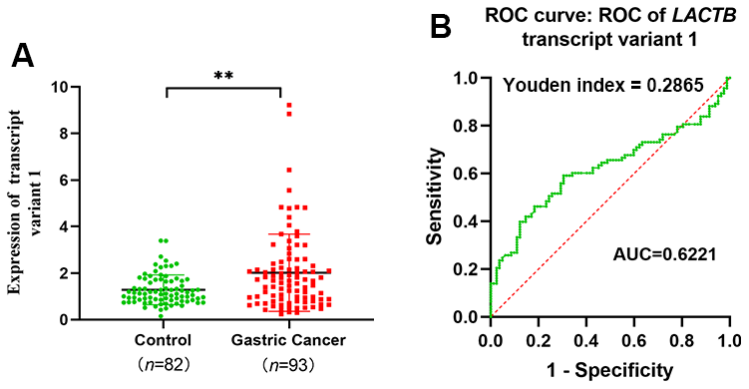
***LACTB* transcript variant 1 expression and diagnosis in peripheral venous blood of gastric cancer patients.** (**A**) Expression level of LACTB transcript 1 in venous peripheral blood of gastric cancer patients*.* (**B**) ROC curve of LACTB transcript variant 1. ^**^P<0.01 vs. Control.

We collected venous peripheral blood from 93 gastric cancer patients and 82 healthy controls to extract RNA and quantify the expression levels of different transcripts of LACTB. In the peripheral venous blood of gastric cancer patients, the expression level of LACTB transcript 1 was significantly higher than that in the healthy controls (P<0.05) (Figure 2A). However, the mRNA quantitative detection results of LACTB transcripts 2 and 3 were very low (Supplementary Figure 1). Furthermore, we performed an ROC curve analysis of LACTB transcript 1 and found that the AUC was 0.6221 (95% CI: 0.5383-0.7059), indicating that LACTB transcript 1 can potentially serve as a biomarker for gastric cancer diagnosis. The maximum Youden index was 0.2865, and the corresponding cut-off value was 1.365. The sensitivity was 0.5914, and the specificity was 0.6951 (Figure 2B). The above findings imply that LACTB transcript 1 is predominantly expressed in the peripheral blood of gastric cancer patients and may serve as a diagnostic marker for gastric cancer. Based on this, subsequent experiments were designed to investigate the impact and underlying mechanisms of altering the expression level of transcript 1 on the biological functions of gastric cancer cells.

### Construction of LACTB transcript 1 overexpressed stable transgenic strain and LACTB knockdown stable transgenic strain

**Figure 3:**
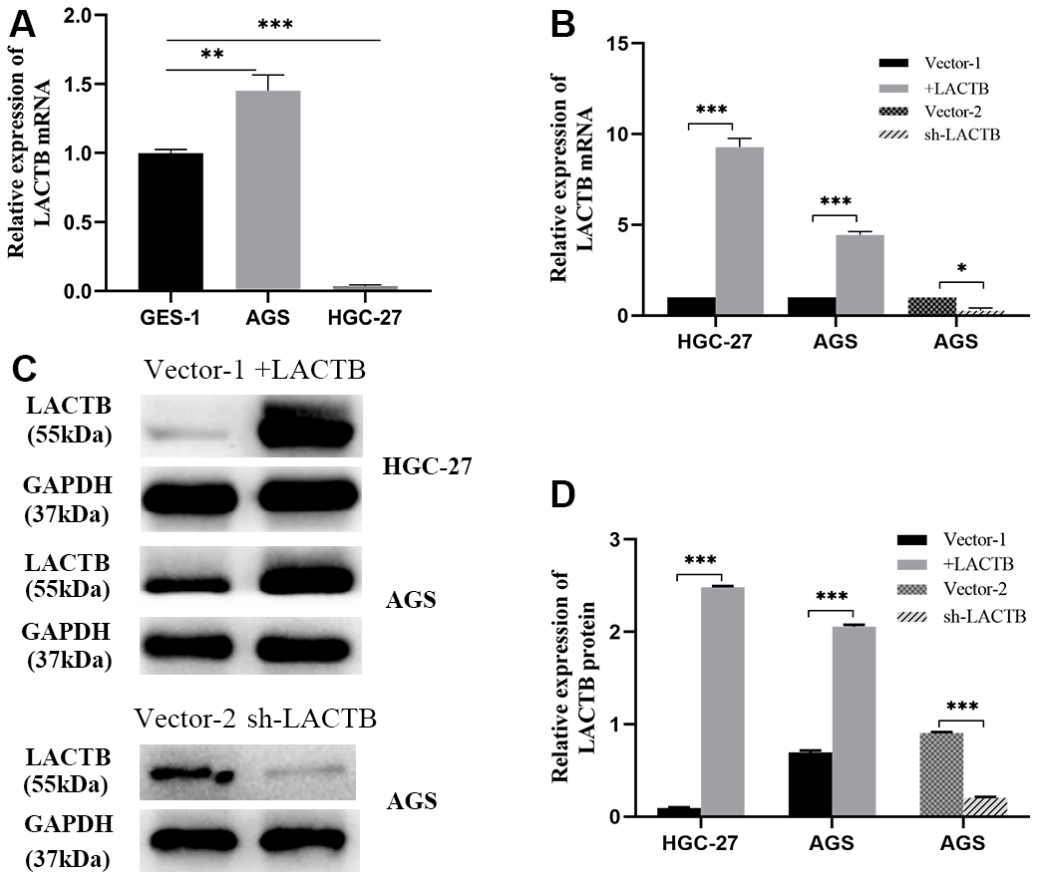
**Construction of LACTB stable overexpression and knockdown cell lines.** (**A**) *LACTB* transcript variant 1 expression in AGS and HGC-27 vs GES-1. (**B**) The expression level of LACTB transcript variant 1 in stably transfected strains. (**C**, **D**) Expression levels of LACTB protein in stably transfected strains. ^*^P<0.05, ^**^P<0.01, ^***^P<0.001.

Compared to the gastric mucosal cell line GES-1, the gastric cancer cell line AGS showed significantly higher expression of LACTB transcript 1, while the undifferentiated gastric cancer cell line HGC-27 exhibited significantly lower expression of LACTB transcript 1 (P<0.05) (Figure 3A). A lentivirus overexpressing LACTB transcript 1 was used to infect AGS and HGC-27 gastric cancer cell lines to establish stable overexpression strains. An empty vector (vector 1) was used to construct corresponding empty cell lines. Meanwhile, the RNAi lentiviral vector was employed to interfere with LACTB expression in AGS, and LACTB-RNAi (67213-1) showed the most prominent knockdown effect. Thus, a stable LACTB knockdown strain was established, and Vector 2 was used to create the corresponding empty cell line. Validation at both the mRNA and protein levels confirmed the successful establishment of stable transgenic strains (P<0.05) (Figure 3B, 3D).

### The influence of LACTB on the migration and invasion abilities of gastric cancer cells

**Figure 4:**
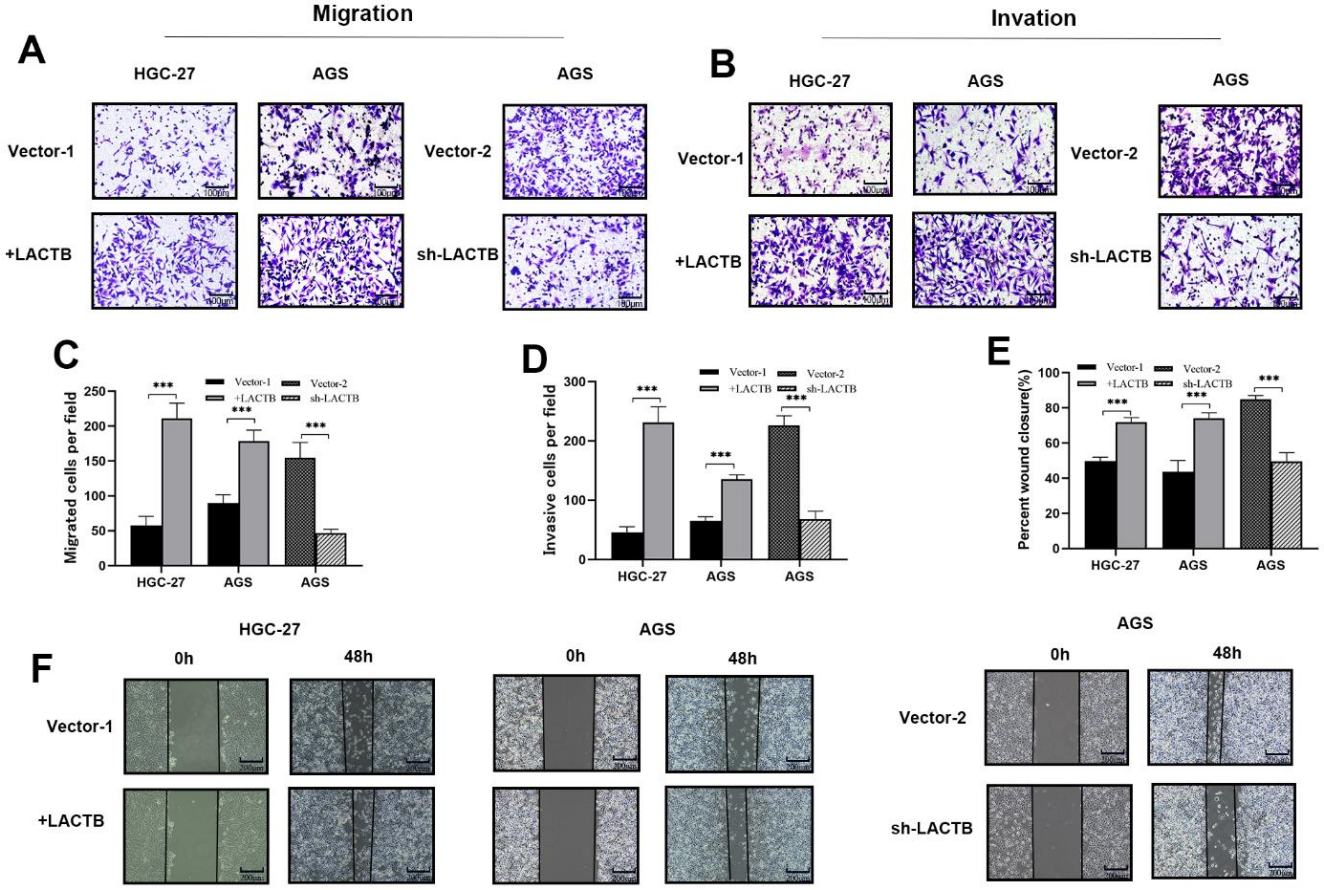
**The effect of LACTB on the migration and invasive ability of gastric cancer cells.** (**A**–**D**) The effect of LACTB on the migration and invasion ability of HGC-27 and AGS. (**E**, **F**) Effects of LACTB on the migration ability of HGC-27 and AGS. ^***^P<0.001 vs. Vector. A, B×200; F×100.

The migration and invasion abilities of gastric cancer cells were examined using Transwell migration and wound healing assays. Additionally, to simulate the human basement membrane, Matrigel was applied to the basement membrane of the Transwell chamber to investigate the effect of LACTB on invasion. The findings demonstrated that gastric cancer cells with upregulated LACTB exhibited a significant increase in the number of cells migrating through the bottom and basement membranes of the Transwell chamber compared to the control group (P<0.001) for both HGC-27 and AGS cell lines (Figure 4A–4D). The wound healing assay indicated that the overexpression of LACTB in HGC-27 and AGS cells resulted in a significantly higher wound closure rate at 48 hours compared to the control group (P<0.001) (Figure 4E, 4F). The downregulated LACTB expression in AGS cells resulted in significantly fewer cells penetrating the basement membrane and a lower wound healing rate compared to the control group (P < 0.001) (Figure 4A–4F). Experimental data indicated that a decreased level of LACTB could impede the migration and invasion of gastric cancer cells.

### The effect of LACTB on EMT of gastric cancer cells

**Figure 5:**
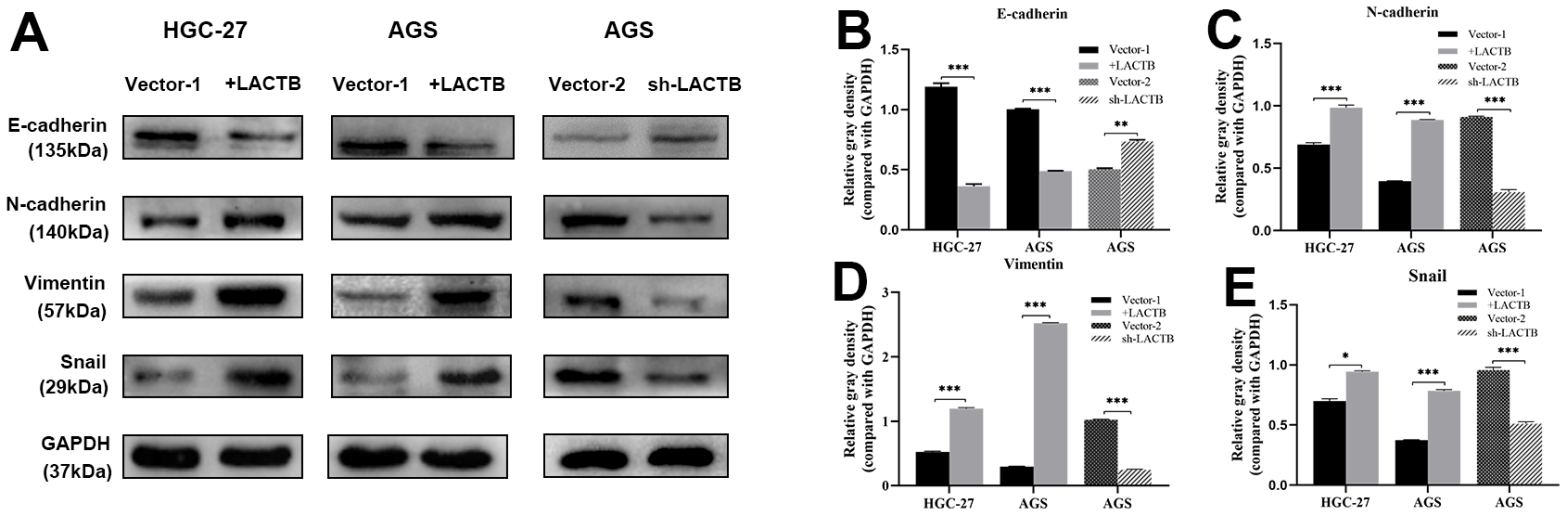
**The effect of *LACTB* on EMT-related proteins in gastric cancer cells.** (**A**) Expression levels of EMT-related proteins in HGC-27-LACTB, AGS-LACTB, AGS-sh-LACTB; (**B**–**E**) Bar graphs of EMT-related protein expression levels; ^*^P<0.05, ^**^P<0.01, ^***^P<0.001 vs. empty group.

The impact of LACTB on the EMT of gastric cancer cells was assessed using Western blot analysis. The results demonstrated that the overexpression of LACTB in HGC-27 and AGS cells led to a decrease in the expression levels of the epithelial marker E-cadherin, while the levels of mesenchymal cell markers, including N-cadherin, Vimentin, and the transcription factor Snail, were upregulated compared to the control group (P<0.05) (Figure 5). AGS-sh-LACTB cells exhibited increased expression of E-cadherin, improved cell adhesion, and decreased expression levels of mesenchymal markers N-cadherin, Vimentin, and Snail, as compared to the corresponding empty vector cells (P<0.05) (Figure 5). The most significant difference was observed in the expression of Vimentin. These results suggest that downregulation of LACTB can inhibit the expression of EMT markers, promote the expression of epithelial markers, and suppress the migration and invasion of gastric cancer cells.

### The effect of LACTB on the level of autophagy in gastric cancer cells

**Figure 6:**
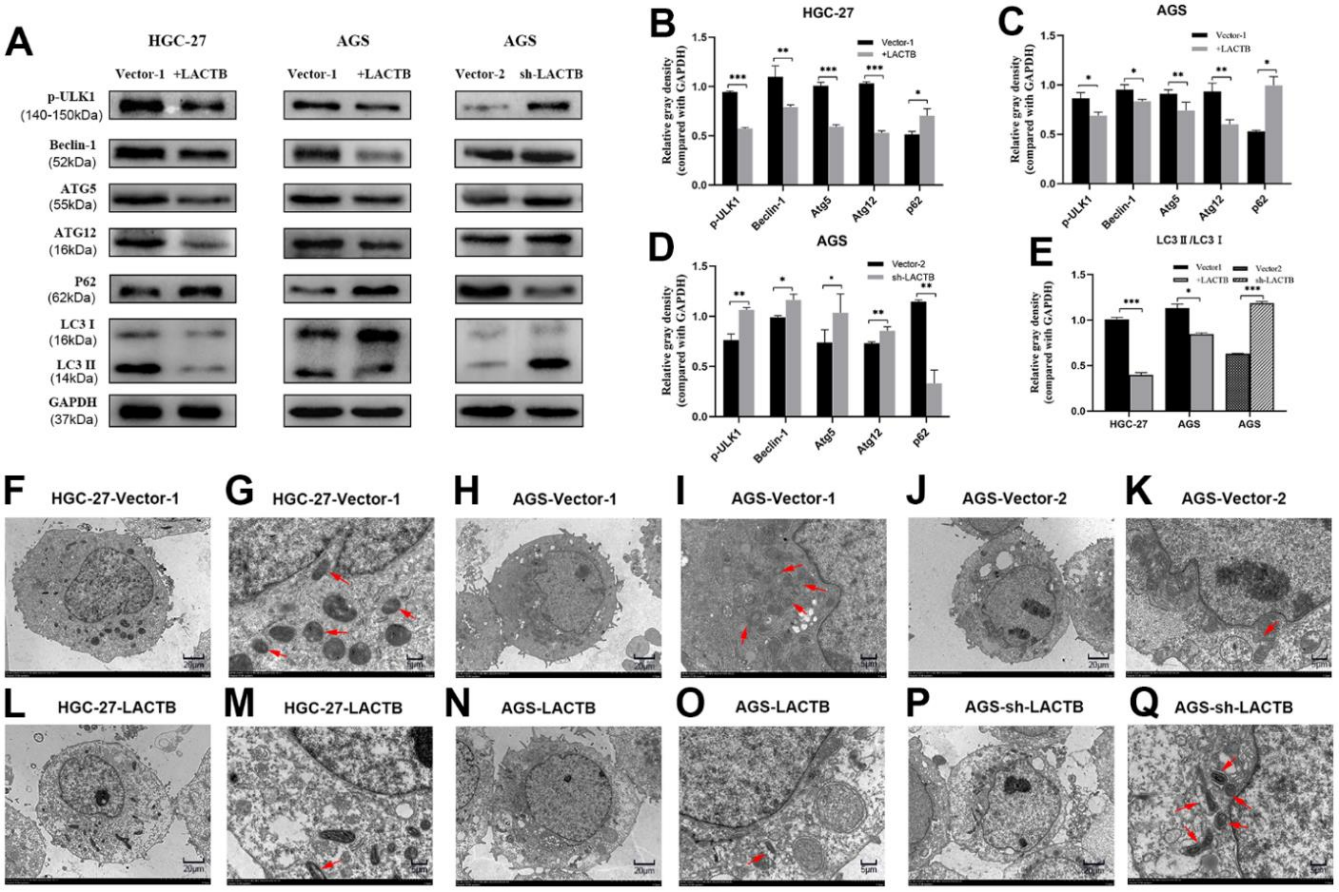
**The effect of LACTB on the autophagy-related proteins in gastric cancer cells.** (**A**) Expression levels of autophagy-related proteins in HGC-27-LACTB, AGS-LACTB, AGS-sh-LACTB; (**B**–**E**) Bar graphs of autophagy-related protein expression levels; (**F**–**Q**) The effect of LACTB on autophagosome formation. The red arrow indicates the autophagosome. (**F**, **H**, **J**, **L**, **N**) P×2.5 k; (**G**, **I**, **K**, **M**, **O**) Q× 8.0 k; ^*^P<0.05, ^**^P<0.01, ^***^P<0.001 vs. empty group.

Immunoblot assay revealed that the overexpression of LACTB in HGC-27 and AGS cells led to a decrease in the expression of key proteins involved in the autophagy signaling pathway, including p-ULK1, Beclin-1, Atg5, and Atg12, as well as a decrease in the LC3II/I ratio. On the other hand, the expression level of P62 was significantly increased (P<0.05) (Figure 6A–6E). On the other hand, knockdown of LACTB led to an increase in the expression levels of key proteins p-ULK1, Beclin-1, Atg5, Atg12, and the ratio of LC3II/I in the autophagy signaling pathway, while the expression level of P62 was significantly decreased (P<0.05) (Figure 6A–6E). Further verification of the effect of LACTB on the autophagy level of gastric cancer cells was carried out using transmission electron microscopy. The results revealed that the number of autophagosomes, characterized by vacuolar bilayer membrane-like structures containing cytoplasmic components, was significantly decreased in HGC-27 and AGS cells overexpressing LACTB compared to the empty group (Figure 6F–6Q). Conversely, knockdown of LACTB in AGS cells significantly increased the number of autophagosomes, as demonstrated by transmission electron microscopy (Figure 6F–6Q). Collectively, these findings provide compelling evidence that LACTB can modulate the autophagy of gastric cancer cells by regulating the autophagy signaling pathway.

## DISCUSSION

LACTB is a recently discovered tumor-related gene that exhibits varying expression levels across different tumor types. In some tumors, LACTB exhibits low expression and functions as a tumor suppressor, which is associated with poor prognosis. These tumors include hepatocellular carcinoma [11], breast cancer [8], colorectal cancer [9, 12, 13], glioma tumor [10], and melanoma [14]. Increased expression of LACTB in these tumors results in the inhibition of tumor cell proliferation, migration, and invasion. In nasopharyngeal carcinoma [15] and pancreatic cancer [16], high LACTB expression has been reported to promote malignant behavior of cancer cells and serve as an unfavorable prognostic indicator. In breast and colorectal cancers, microRNAs mainly negatively regulate the expression of LACTB [8, 13]. In colorectal cancer cells, upregulation of LACTB expression can inhibit the cellular EMT process and increase the expression of epithelial markers through the autophagy signaling pathway involving PI3K/AKT/mTOR [12]. However, there are certain tumors where LACTB is expressed at a high level, such as in nasopharyngeal carcinoma. In this context, silencing LACTB can inhibit the metastasis of nasopharyngeal carcinoma by inhibiting ERBB3/EGFR signal transduction and increasing the stability of histone H3 [15]. The above studies demonstrate that LACTB’s functional roles LACTBin different tumor types are inconsistent and may even exhibit opposing effects. This discrepancy could stem from the diverse mechanisms through which LACTB functions in various tumor types.

UALCAN analysis indicated a marked increase in LACTB expression levels within gastric cancer tissues compared to normal controls. This observation was supported by results obtained from peripheral blood experiments which revealed a significant upregulation of LACTB transcript variant 1 expression among gastric cancer patients relative to healthy individuals. These experimental results were in agreement with those obtained from prior bioinformatics analyses. Furthermore, the peripheral blood expression data for both LACTB and its transcript were utilized to generate a ROC curve. This analysis indicated that LACTB transcript 1 may possess diagnostic value for gastric cancer. Taken together, these findings suggest that LACTB may play a role in the pathogenesis and progression of gastric cancer, potentially serving as an adverse prognostic factor for this disease. Establishing a reference range for transcript variant 1 expression levels in healthy individuals may facilitate the detection of LACTB transcript variant 1 expression in peripheral blood of gastric cancer patients, potentially aiding in disease diagnosis. The incorporation of this marker in conjunction with existing tumor markers could enhance the diagnostic accuracy and monitoring of gastric cancer, thus contributing to the development of non-invasive diagnostic indicators for this disease.

Furthermore, enrichment analysis of LACTB-related genes indicated potential involvement in cellular immunity, cell adhesion, and lysosome, a key component of cellular autophagy [18]. Both cellular immunity [36] and adhesion have been shown to play integral roles in the EMT of gastric cancer cells. Given these observations, it is highly conceivable that LACTB may modulate the invasive and migratory capacity of gastric cancer cells via regulation of autophagy and EMT. Consequently, additional experiments could be designed to investigate LACTB expression in gastric cancer and its impact on the biological behavior of cancer cells, as well as to assess whether LACTB may regulate EMT through modulation of autophagy, thereby exerting effects on the biological function of gastric cancer cells. Current research findings suggest that LACTB can modulate the migration and invasion capacity of cancer cells in multiple solid tumors, although it has been observed to exhibit inhibitory effects in some tumors [8, 9, 11] and oncogenic effects in others [15]. The present study aimed to investigate the impact of LACTB expression modulation on the migration and invasion potential of AGS and HGC-27 gastric cancer cells. Results demonstrated that upregulation of LACTB in AGS and HGC-27 gastric cancer cells promoted the migratory and invasive behavior of gastric cancer cells. Inhibition of LACTB expression in AGS cells resulted in a reduction in the invasive and migratory capacities of gastric cancer cells. Accordingly, upregulation of LACTB expression promotes the migratory and invasive potential of gastric cancer cells, while downregulation of LACTB expression impedes these abilities, consistent with previous investigations on LACTB in nasopharyngeal carcinoma [15]. Indeed, attenuation of LACTB expression levels could impede the proliferation, migration, and invasion of gastric cancer cells. This provides the foothold for exploring therapeutic approaches aimed at restraining the growth and metastasis of gastric cancer cells.

Subsequent experiments aimed at elucidating the underlying mechanisms through which LACTB promotes the migratory and invasive capabilities of gastric cancer cells revealed that overexpression of LACTB in HGC-27 and AGS cells could enhance the EMT in these cells, while silencing LACTB impeded the EMT process in gastric cancer cells. Our study findings demonstrated that induction of LACTB overexpression led to alterations in EMT-specific markers, as well as a decrease in the levels of autophagy-related molecules, particularly the activated LC3II protein within and outside autophagosomes, and a significant reduction in the number of autophagosomes. Increased expression levels of the “linker” molecule P62 have been associated with diminished selective uptake and degradation of autophagic substrates. Earlier investigations have also highlighted that autophagy can be modulated by EMT-associated signaling pathways [26]. These findings suggest a correlation between EMT and autophagy levels in the current study, with elevated autophagy levels being advantageous in restraining the progression of gastric cancer. In summary, upregulated expression of LACTB has been shown to prompt gastric cancer cells to adopt the EMT phenotype by impeding autophagy levels. Conversely, low levels of LACTB have been observed to increase autophagy levels, which indirectly hinders EMT of gastric cancer cells and subsequently diminishes their migratory and invasive potential. These results suggest that inhibition of LACTB expression can prevent EMT by inducing autophagy. Overall, it may represent a novel strategy for anti-cancer therapy to regulate autophagy levels, with the aim of reducing EMT.

## CONCLUSIONS

The findings from this investigation reveal that LACTB is upregulated in gastric cancer and may serve as a molecular marker for disease diagnosis. Additionally, the expression level of LACTB transcript variant 1 can significantly affect the migratory and invasive potential of gastric cancer cells. Subsequent research findings suggested that LACTB may attenuate EMT progression via modulation of autophagy levels, leading to inhibition of gastric cancer cell migration and invasion. These results suggest that LACTB may interfere with gastric cancer metastasis by modulating autophagy levels, thus providing a potential experimental basis for investigating the underlying mechanism of LACTB in late-stage gastric cancer.

## MATERIALS AND METHODS

### Cell culture

Human gastric cancer cell lines GES-1, AGS, and HGC-27 were procured from the Cell Bank of the Chinese Academy of Sciences. The cells were cultured under standard conditions in a complete medium supplemented with 10% fetal bovine serum (Gibco, USA) and maintained in a culture environment with 5% CO_2_ at 37° C. The cell growth status was monitored using an inverted microscope, and the cells were subcultured every 2-3 days. The cells in the logarithmic growth phase were harvested for the subsequent experiments.

### Venous peripheral blood sample

We collected peripheral venous blood samples from 93 patients with gastric cancer from the Affiliated Hospital of Guizhou Medical University. All patients were diagnosed through pathological examination from April 2021 to December 2021, and 82 healthy individuals were collected as controls during the same period. The collection of peripheral blood samples in this study was in compliance with the ethical standards formulated by the Ethics Committee (Approval No: 2021 Ethics No. 337 of the Ethics Committee for Human Trials, Guizhou Medical University). The detailed clinical information of patients was shown in Supplementary Table 1.

### Bioinformatics analysis

Information on LACTB was extracted from NCBI’s database, LACTB (GeneID: 114294) and a preliminary sequence analysis was conducted. The expression levels of LACTB total mRNA in gastric cancer were examined using Oncomine and UALCAN analysis websites. The LinkFinder module of LinkedOmics was used to analyze the differentially expressed genes associated with LACTB and GO and KEGG enrichment analysis was conducted using DAVID to elucidate the potential biological pathways and processes associated with LACTB-related genes.

### Design of specific primers for LACTB gene

NCBI database analysis revealed that LACTB (GeneID: 114294) consists of 7 exons and 3 standard mRNAs annotated as NM sequences: transcript 1 (V1, NM_032857.5), transcript 2 (V2, NM_171846 .4), and transcript 3 (V3, NM_001288585.2), as well as 2 non-coding mRNAs annotated as XR sequences, XR1 (XR_931745.2) and XR2 (XR_429442.2). To conduct RT-qPCR experiments, a reverse primer was designed at the unique exon 6 of transcript 1. The Table 1 below displays the primers used in the experiment.

**Table 1:** Primer sequences for RT-qPCR.

**Name**	**Primer sequence**	
*LACTB* transcript variant 1	F1092	CAGGAAGAAAACGAGCCAGTG
	R1230	GGTCACCCACTGTAGACAGAA
*LACTB* transcript variant 2	F945-2	CCTTTGTTCTTCAAACCTGGTAG
	R1158	GCTAGACACAGCAGAAGGTA
*LACTB* transcript variant 3	F945-3	CCTTTGTTCTTCAAACCTGTCA
	R1158	GCTAGACACAGCAGAAGGTA
*HPRT*	F148	ATGGCGACCCGCAGCCCT
	R266	CCATGAGGAATAAACACCCT

### Real-time fluorescent quantitative PCR reaction

Total RNA was extracted from gastric cancer cell lines and peripheral blood samples using the UNIQ-10 column Trizol total RNA extraction kit (Sangon Biotech, B511321-0100) in accordance with the manufacturer’s instructions. The extracted RNA was then reverse transcribed into cDNA using the PrimeScript™ RT reagent Kit with gDNA Eraser (Takara, RR047A). TB Green® Premix Ex Taq™ II (Tli RNaseH Plus) (Takara, RR820A) was employed for RT-qPCR analysis, with HPRT serving as an internal reference gene for determining the relative expression level of LACTB. The data were analyzed via the 2^-ΔΔCt^ method to evaluate the relative expression of the target gene.

### Western blotting

To extract the total protein, 80 μl of PMSF-containing lysis buffer was added to each well of a 6-well plate, and the cells were lysed on ice for approximately 30 minutes. The concentration of the extracted protein was determined using the BCA kit (Solarbio, China). During SDS-PAGE electrophoresis, 30 μg of total protein was loaded into each well. The specific primary antibody was utilized to detect the corresponding protein, and the image was captured with a Bole blot exposure apparatus. Subsequently, the integrated optical density value of the protein band was determined using image J analysis software. In each sample, the GAPDH protein band was also detected as an internal control. The primary antibodies used in the experimental procedure included E-Cadherin (3195), N-Cadherin (13116T), Snail (3879T), Vimentin (5741T), LC3A/B (4108S), Atg5 (12994T), Atg12 (2010), p-ULK1 (14202T), which were purchased from Cell Signaling Technology, USA. Additionally, SQSTM1/p62 (ab109012), Beclin-1 (ab210498), and LACTB (ab131171) were purchased from Abcam.

### Overexpression of lentiviral vector to construct the LACTB overexpression stable strain

The gene of interest, transcript 1 of LACTB (NM_032857.5), was inserted into the Ubi-MCS-SV40-EGFP-IRES-puromycin vector (accession GV367, Gene Kai). To generate stable cell lines, gastric cancer cell lines AGS and HGC-27 were infected with lentivirus containing LACTB overexpression construct or empty vector control. The resulting cell lines included HGC-27 empty cell line (HGC-27-Vector) and HGC-27LACTB overexpressing stable strain (HGC-27-LACTB), as well as AGS empty cell line (AGS-Vector) and AGS-LACTB overexpression stable strain (AGS-LACTB). To select cells with 100% infection efficiency, puromycin (2 μg/ml) was added to the culture medium.

### RNAi lentiviral vector to construct LACTB knockdown stable strain

The siRNA sequence (LACTB-RNAi-67213-1: GAGCAGGAGAATGAAGCCAAA) targeting LACTB transcript 1 (NM_032857.5) was inserted into the hU6-MCS-CBh-gcGFP-IRES-puro-mycin vector (number GV493, GENECHEM Inc.). AGS cells were then infected with the resulting construct to generate a LACTB knockdown strain (AGS-sh-LACTB) and an empty vector control stable strain (AGS-Vector-shRNA). Puromycin (2 μg/ml) was added to the culture medium to select cells with 100% infection efficiency.

### Wound healing experiment

During the experiment, 2 ml of a cell suspension containing 8 × 10^5^ cells was added to each well of a six-well culture plate. The cells were then incubated for 24 hours in a medium containing 10% fetal bovine serum. Afterward, using a 200 μl pipette tip, a “cross”-shaped scratch was created on the cell surface of the dish. The old medium was aspirated and replaced with media containing 1% fetal bovine serum. The cells were then cultured in a 5% CO_2_ incubator at 37° C. Images (100×) were collected at 0 h, 24 h, and 48 h to measure the width of the scratch, and the scratch healing rate was calculated using Image J.

### Cell invasion/migration experiment

In accordance with standard laboratory practices, the pre-cooled 1640 medium was subjected to Matrigel (200μg/ml; Corning Costar, USA). Sixty microliters of this mixture were added to the upper chamber for solidification, and any excess or uncoagulated gel was subsequently removed. To introduce a total of 80,000 cells, a volume of 200 μl of cell suspension was added to the upper chamber. Meanwhile, 750 μl of medium containing 20% FBS was added to the lower chamber. Notably, the medium utilized for migration assays contained only 10% FBS, and the chamber was not coated with Matrigel. The cell chamber was then incubated under standard conditions of 37° C and 5% CO_2_ for a predetermined duration. After incubation, the cell chamber was removed, and 4% paraformaldehyde was employed to fix the cells for 30 minutes. Subsequently, the cells were stained with 0.1% crystal violet for 10 minutes, and the cells located on the upper layer of the filter membrane were wiped off using a cotton swab before being left to air-dry. In accordance with established protocols, the cells present in the lower filter layer were meticulously examined via a high-powered microscope. Subsequently, 5 distinct and randomly selected fields of view were meticulously assessed, and the photographs (200×) obtained were meticulously analyzed to extract the average values. The AGS cells in the LACTB overexpression group were subjected to an exhaustive 24-hour incubation process at 37° C for the invasion assay and a 20-hour incubation process for the migration assay. Conversely, the AGS cell LACTB knockdown group was subjected to a 30-hour incubation process at 37° C for the invasion assay and a 24-hour incubation process for the migration assay. Similarly, the HGC-27 cells in the LACTB overexpression group were incubated for 48 hours at 37° C for the invasion assay and 30 hours for the migration assay.

### Transmission electron microscopy (TEM)

Once the cells reached a confluence of 80% in the culture flask, the culture medium was removed and replaced with an electron microscope fixative. The cells were then fixed at 4° C for 2-4 hours before being centrifuged at low speed to obtain mung bean-sized cell clumps. The cell clumps were subsequently pre-embedded with 1% agarose, post-fixed with 1% osmic acid for 2 hours, and then dehydrated with alcohol at room temperature. After dehydration, the cell clumps were embedded with acetone and 812 embedding medium and polymerized in an oven at 60° C for 48 hours. Ultramicrotome slices, 60-80 nm thick, were then obtained. Uranium-lead double staining, which involved the use of a 2% uranyl acetate saturated alcohol solution and lead citrate, was performed for 15 minutes each, and the sections were left to dry overnight at room temperature. The samples were then observed under a transmission electron microscope, and images were collected for further analysis. Autophagosomes, characterized by a double membrane enveloping a portion of the cytoplasm, were identified. These autophagosomes break down various cytoplasmic components, including mitochondria and fragments of the endoplasmic reticulum, through fusion with lysosomes to degrade their contents.

### Statistical analysis

Data analysis was performed using SPSS 19.0 (SPSS, Chicago, USA) and GraphPad Prism 8.0 software. For normally distributed measurement data, the grouped independent samples t-test was utilized and expressed as mean ± standard deviation (SD). A P-value less than 0.05 was statistically significant.

### Data availability statement

The original contributions presented in the study are included in the article Supplementary Material. We have uploaded all my source code and experimental data to a cloud disk (https://www.jianguoyun.com/p/DQXDaewQhv2xCxiI7PUEIAA). Further inquiries can be directed to the corresponding author.

## Supplementary Material

Supplementary Figure 1

Supplementary Table 1

Supplementary Table 2
