# Supplementary material for: A potential therapeutic approach for gastric cancer: inhibition of LACTB transcript 1
Source: Aging (Albany NY). 2023 Dec 26;15(24):15213–27. doi: 10.18632/aging.205345 (PMC10781463; doi:10.18632/aging.205345)
Supplement: Supplementary Figure 1 [file aging-15-205345-s001.pdf]

SUPPLEMENTARY FIGURE

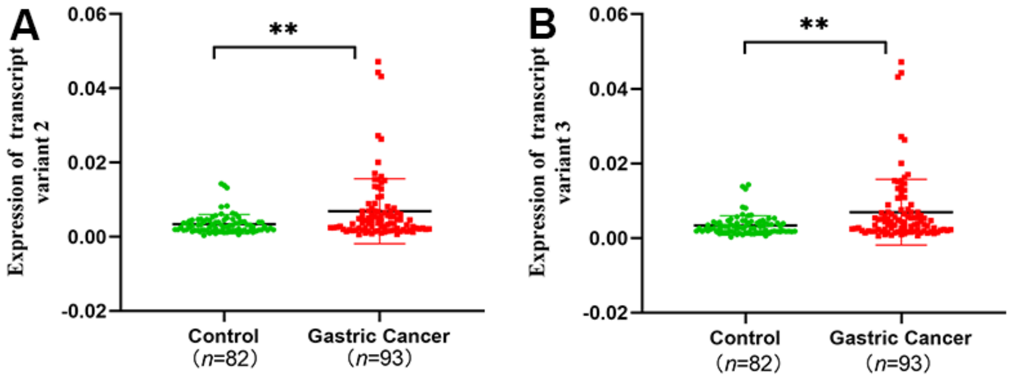

Supplementary Figure 1. (**A**) Expression level of LACTB transcript 2 in venous peripheral blood of gastric cancer patients; (**B**) Expression level of LACTB transcript 3 in venous peripheral blood of gastric cancer patients. $^{**}P<0.01$ vs Control.
